# Reliability and Variability of Lower Limb Muscle Activation as Indicators of Familiarity to Submaximal Eccentric Cycling

**DOI:** 10.3389/fphys.2022.953517

**Published:** 2022-07-08

**Authors:** Joel A. Walsh, Darryl J. McAndrew, Jonathan Shemmell, Paul J. Stapley

**Affiliations:** ^1^ Neural Control of Movement Laboratory, School of Medical, Indigenous and Health Sciences, Faculty of Science, Medicine and Health, University of Wollongong, Wollongong, NSW, Australia; ^2^ Graduate School of Medicine, Faculty of Science, Medicine and Health, University of Wollongong, Wollongong, NSW, Australia; ^3^ Neuromotor Adaptation Laboratory, School of Medical, Indigenous and Health Sciences, Faculty of Science, Medicine and Health, University of Wollongong, Wollongong, NSW, Australia

**Keywords:** electromyography, learning, lengthening contraction, moderate load, negative work, pedaling

## Abstract

Submaximal eccentric (ECC) cycling exercise is commonly used in research studies. No previous study has specified the required time naïve participants take to familiarize with submaximal ECC cycling. Therefore, we designed this study to determine whether critical indicators of cycling reliability and variability stabilize during 15 min of submaximal, semi-recumbent ECC cycling (ECC cycling). Twenty-two participants, aged between 18–51 years, volunteered to complete a single experimental session. Each participant completed three peak eccentric torque protocol (PETP) tests, nine countermovement jumps and 15 min of submaximal (i.e., 10% peak power output produced during the PETP tests) ECC cycling. Muscle activation patterns were recorded from six muscles (rectus femoris, RF; vastus lateralis, VL; vastus medialis, VM; soleus, SOL; medial gastrocnemius, GM; tibialis anterior, TA), during prescribed-intensity ECC cycling, using electromyography (EMG). Minute-to-minute changes in the reliability and variability of EMG patterns were examined using intra-class correlation coefficient (ICC) and variance ratios (VR). Differences between target and actual power output were also used as an indicator of familiarization. Activation patterns for 4/6 muscles (RF, VL, VM and GM) became more consistent over the session, the RF, VL and VM increasing from moderate (ICC = 0.5–0.75) to good (ICC = 0.75–0.9) reliability by the 11th minute of cycling and the GM good reliability from the 1st minute (ICC = 0.79, ICC range = 0.70–0.88). Low variability (VR ≤ 0.40) was maintained for VL, VM and GM from the 8th, 8th and 1st minutes, respectively. We also observed a significant decrease in the difference between actual and target power output (χ^2^_14_ = 30.895, *p* = 0.006, *W* = 0.105), expressed primarily between the 2nd and 3rd minute of cycling (*Z* = -2.677, *p* = 0.007). Indicators of familiarization during ECC cycling, including deviations from target power output levels and the reliability and variability of muscle activation patterns stabilized within 15 min of cycling. Based upon this data, it would be reasonable for future studies to allocate ∼ 15 min to familiarize naïve participants with a submaximal ECC cycling protocol.

## Introduction

Eccentric (ECC) cycling is a novel task that involves applying an opposing resistance to backward-rotating motor-driven pedals (Walsh et al., 2021a) and, compared to concentric cycling, requires distinctly different muscle activation patterns (Peñailillo et al., 2013; Clos and Lepers, 2020; Ema, 2022). To overcome the novelty of ECC cycling participants require a period of practice or familiarization (Green et al., 2017; Nosaka et al., 2017; Penailillo et al., 2017; Kan et al., 2019).

Previous suggestions of unfamiliarity impacting ECC cycling coordination (Green et al., 2017; Penailillo et al., 2017) are not unexpected given that unfamiliarity of a novel task adversely affects neuromuscular control (Matsas et al., 2000; Bischoff et al., 2012; Walsh et al., 2019). Subsequently, it is reasonable to hypothesize that any learning effect, associated with novel ECC cycling (LaStayo et al., 2008; Purtsi et al., 2012; Brughelli and Van Leemputte, 2013; Kan et al., 2019), would similarly affect neuromuscular control of muscle activation patterns recorded from naïve participants, during ECC cycling. Indeed, variable muscle activation patterns recorded during cycling correlates with increased physiological cost and reduced efficiency (Waldron et al., 2016). Reducing variability of muscle activation patterns by familiarizing participants with a novel task (i.e., ECC cycling) (Osu et al., 2002; Calder et al., 2005; Waldron et al., 2016), would improve the reliability of physiological (i.e., metabolic cost and efficiency) (Huang et al., 2012; Waldron et al., 2016) and neuromuscular control (Matsas et al., 2000; Bischoff et al., 2012) measures recorded during ECC cycling and thereby, improving interpretation of findings. Therefore, it would seem essential to understand if ECC cycling requires a defined period of familiarization to achieve consistent muscle activation patterns.

Previously reported familiarization protocols vary considerably in length. For example, studies have used single, short-duration (5 minutes) periods of practice to familiarize participants with ECC cycling at low intensity ( ∼ 50 Watts or 10–15% peak concentric torque) (Peñailillo et al., 2015; Penailillo et al., 2017; Rakobowchuk et al., 2018; Kan et al., 2019). Alternatively, others have assumed familiarization occurs following up to 15 min (min) of ECC cycling (Pageaux et al., 2020; Clos et al., 2022). However, it is unknown whether the aforementioned protocols adequately familiarize naïve participants with ECC cycling. Moreover, to our knowledge, only two studies have investigated familiarization to semi-recumbent ECC cycling. These studies have focused on familiarization to maximal (Green et al., 2017) and submaximal (Clos and Lepers, 2020) ECC cycling across multiple, short-duration sessions (i.e., 10–90 s). These protocols are applicable to maximal ECC cycling or ECC cycling training studies. However, there is no current protocol for determining familiarization to submaximal, longer-duration (i.e., >10 min) ECC cycling, despite being commonly used in research studies (Peñailillo et al., 2015; Penailillo et al., 2017; Rakobowchuk et al., 2018; Kan et al., 2019).

Developing a single session familiarization protocol of adequate duration (>10 min) (Zych et al., 2018) and intensity (Walsh et al., 2021b) could be better used to familiarize participants with ECC cycling. Furthermore, single session protocols, as opposed to multiple visit protocols, reduce time constraints that could affect participation (Martin et al., 2007; Thompson et al., 2016). Therefore, this study aimed to determine if naïve participants familiarize with submaximal, semi-recumbent ECC cycling (ECC cycling) within a single 15-min session. A 15 min cycling duration was considered sufficient to determine single-session familiarization to ECC cycling based on 1) previous findings suggesting that at least 10 min of cycling is required to adapt to novel cycling (asynchronous cycling) (Zych et al., 2018), 2) similar ECC cycling durations (10–20 min) adopted in past studies (Peñailillo et al., 2015; Penailillo et al., 2017; Kan et al., 2019) and, 3) that ECC exercise protocols lasting between 5–30 min are considered moderate load (i.e., submaximal intensity) (Hoppeler and Herzog, 2014; Hoppeler, 2016). Reliability and variability of lower limb muscle recruitment patterns, measured using surface electromyography, were interpreted as indicators of familiarity, given that increased reliability and decreased variability of measured variables are consistent with improved task execution following repeated performance and familiarization (Hopkins et al., 2001; Brughelli and Van Leemputte, 2013; Sampson et al., 2013; Higgins et al., 2014). It was hypothesized that naïve participants will adequately familiarize with ECC cycling during a single 15-min session by producing reliable muscle activation patterns of low variability, while maintaining a controlled workload.

## Materials and Methods

### Participants

Twenty-two healthy participants aged between 18–51 years (age = 32 ± 9 years; height = 180.1 ± 7.9 cm; mass = 75.5 ± 12.2 kg^−1^) volunteered to participate in this study. Participants had no previous ECC cycling experience and completed a pre-screening questionnaire to determine exercise readiness (Sports Medicine Autralia, 2005). All experimental procedures described in this study were granted ethical approval by the University’s Human Research Ethics Committee (ethics number 2019/438) and carried out in accordance with the Declaration of Helsinki (World Medical Association, 2013). Written informed consent was obtained from each participant. Participants were asked to refrain from consuming caffeine (12 h), alcohol (24 h) prior to testing and strenuous physical activity, on the day, prior to testing.

### Experimental Protocol

**FIGURE 1:**
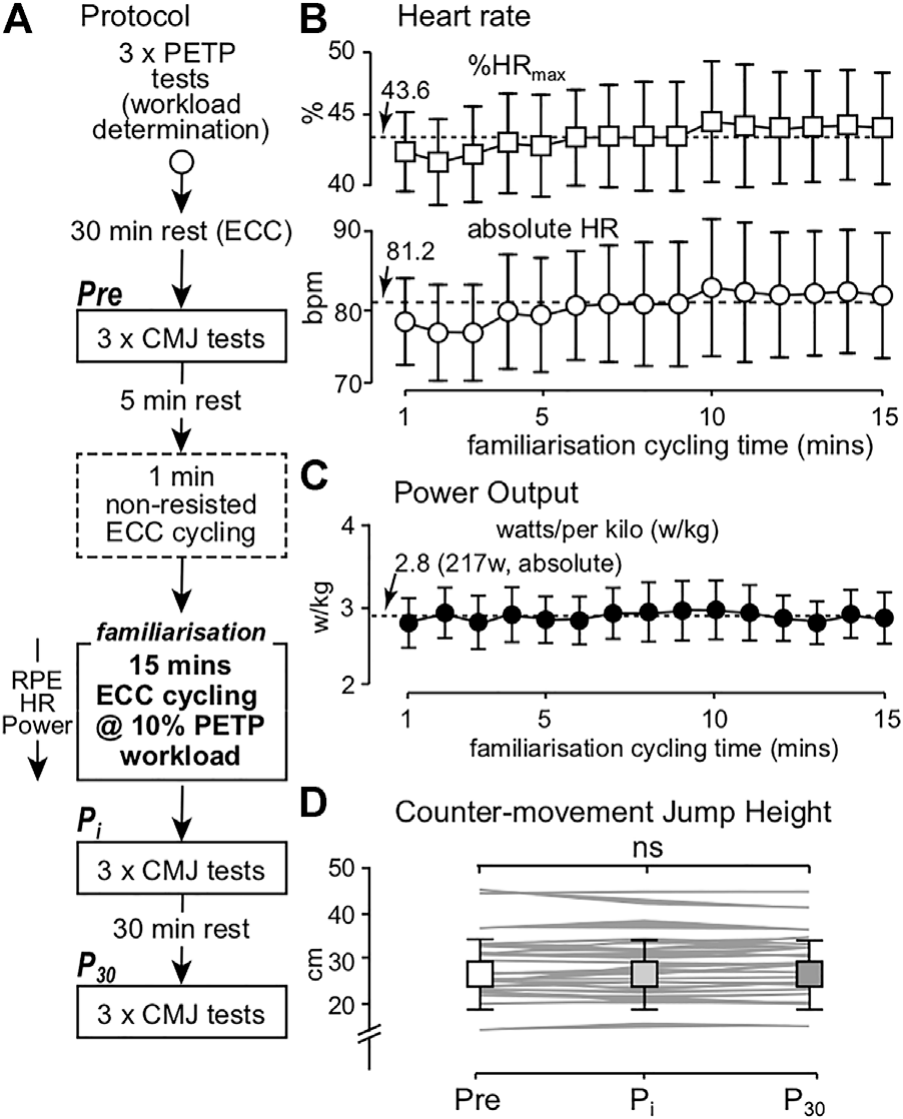
**(A)** Experimental protocol; **(B)** group mean (±95% CI, *n* = 22) %HR_max_ (squares) and absolute HR (bpm, circles) values; **(C)** group mean (±95% CI) relative power output recorded from 21 participants (due to the power file of one participant not saving post ECC cycling); **(D)** group mean (±SD) and individual mean (greyscale lines) CMJ height values recorded pre- (Pre), immediately post (P_i_) and 30-min post (P_30_) ECC cycling. Broken lines represent the overall mean %HR_max_, absolute HR and relative power output recorded during ECC cycling. ns denotes no significant difference.

Participants completed a single experimental visit designed to determine within-session familiarization, based on variability and reliability of muscle EMG patterns, to ECC cycling. Participants performed three peak eccentric torque protocol (PETP) tests (Walsh et al., 2021b), nine countermovement jumps (CMJ) and 15 min of ECC cycling (Figure 1A). Prior to ECC cycling, each participant performed 1 minute of non-resisted ECC pedaling (i.e., freewheeling) to become aware of the motion induced by the cycle ergometer.

Eccentric cycling was conducted on a custom-modified semi-recumbent ECC cycle ergometer (Walsh et al., 2021a). Participants were instructed to perform ECC cycling by only resisting the backwards rotating pedals when opposable (i.e., 260–360°) and to passively follow the pedals when non-opposable (Walsh et al., 2021a). This angle—260–360°—corresponds to the opposable phase of an ECC pedal cycle, where participants are able to apply an opposable resistive force to the pedal, resulting in an ECC contraction of the exercising muscles (Walsh et al., 2021a). Power output, cadence and cycling time were continuously recorded during ECC cycling and displayed on a touchscreen monitor. Cadence was fixed at 60 rpm. Eccentric cycling intensity was prescribed at 10% of peak power output obtained during the PETP tests (Walsh et al., 2021b). The prescribed ECC cycling intensity was calculated from peak power output values recorded during the PETP test. The PETP test is a recently developed test where participants apply a maximal ECC resistive force to a backwards moving pedal arm, fixed to an isokinetic dynamometer (Walsh et al., 2021b). The PETP test replicates the position (i.e., semi-recumbent), speed (60 rpm) and phase of an ECC pedal cycle (Walsh et al., 2021b). Participants were instructed to closely match their real-time power output with that prescribed (i.e., target) using their 10% peak PETP test value. Heart rate (HRM-dual™, Garmin Ltd., Schaffhausen, Switzerland) was measured continuously during ECC cycling and ratings of perceived exertion (RPE), perceived effort and muscle soreness scores recorded per minute. Age-predicted maximal heart rate (HR_max_) was calculated using a previously validated equation (HR_max_ = 208—(0.7 × age) (Tanaka et al., 2001). RPE as recorded using a Borg 6–20 scale and indicated as the *‘degree of heaviness and strain experienced during physical work’* (Penailillo et al., 2017) relating to whole-body exertion. Perceived exertion and muscle soreness were recorded using a 100 mm visual analog scale and indicated *‘the amount of mental or physical energy being given to a task’* and level of pain within the quadriceps during ECC cycling, respectively (Penailillo et al., 2017). Heart rate, RPE, perceived exertion and muscle soreness data were used as secondary measures of ECC cycling intensity.

### Countermovement Jump

Countermovement jump tests (3 ×  3 repetitions) were conducted pre-, immediately post and 30-min post ECC cycling (Figure 1A) to monitor neuromuscular status (Claudino et al., 2017), as an objective measure of lower limb fatigue (Sanchez-Medina and González-Badillo, 2011; Balsalobre-Fernández et al., 2014). Frontal video footage of all CMJ tests were recorded on an iPad Air 2 (Version 13.3.1, Apple Inc., USA) using a mobile application (*My Jump* 2, Version 4.2 iOS application for Mac, Apple Inc., USA) (Balsalobre-Fernández et al., 2015). Flight time between the take-off and landing frames was used to determine CMJ jump height (Balsalobre-Fernández et al., 2015). Maximal jump height was calculated for each CMJ and averaged per collection time for analysis of neuromuscular status.

### Surface Electromyography

Surface electromyography (EMG) was recorded from six muscles (rectus femoris, RF; vastus lateralis, VL; vastus medialis, VM; soleus, SOL; medial gastrocnemius, GM; tibialis anterior, TA) of the dominant leg (Zych et al., 2018) using 10 mm diameter Ag/AgCl bipolar electrodes (Bagnoli™, Delsys Incorporated, Natick, MA, USA). These muscles were selected based on their involvement in semi-recumbent cycling (Hakansson and Hull, 2005). More specifically, RF, VL, VM and GM were considered as the primary active muscles, used during ECC cycling, due to the majority of power absorption occurring at the knee (58%) and ankle (10%) joints through knee extension (i.e., RF, VL and VM) and plantar flexion (i.e., GM) (Elmer et al., 2010; Green et al., 2017; Penailillo et al., 2017). Electrode sites were prepared by shaving, mildly abrading, and cleansing the skin with isopropyl alcohol to improve electrode-skin contact (Merletti and Di Torino, 1999). Electrodes were positioned over the muscle belly and parallel to the direction of the respective muscle fibers, by the same researcher, in accordance with the recommendations by Surface Electromyography for Non-Invasive Assessment of Muscles (SENIAM guidelines) (Hermens et al., 2000). The reference electrode was fixed over the right clavicle.

Raw EMG signals were sampled at 2,000 Hz, gain amplified ( × 1,000), digitized using a 16-bit analogue-to-digital converter (Power1401, Cambridge Electronic Design, Cambridge, UK) and exported for offline analysis. Offline analysis was performed using Spike software version 6.02 (Cambridge Electronic Design, Cambridge, UK). EMG signals were full-wave rectified, DC-offset and band pass filtered between 10 (high pass) and 500 (low pass) Hz using a 4th order low-pass Butterworth filter (high pass 0.5 dB and low pass 20 dB) (Chapman et al., 2008; Hug et al., 2008). EMG data were smoothed using a root mean square (RMS_EMG_) algorithm calculated over consecutive pedal cycles using a 25-millisecond moving average window for each muscle activation pattern (Hug et al., 2008).

### Data Analysis

#### Processing EMG Muscle Activation Patterns

**FIGURE 2:**
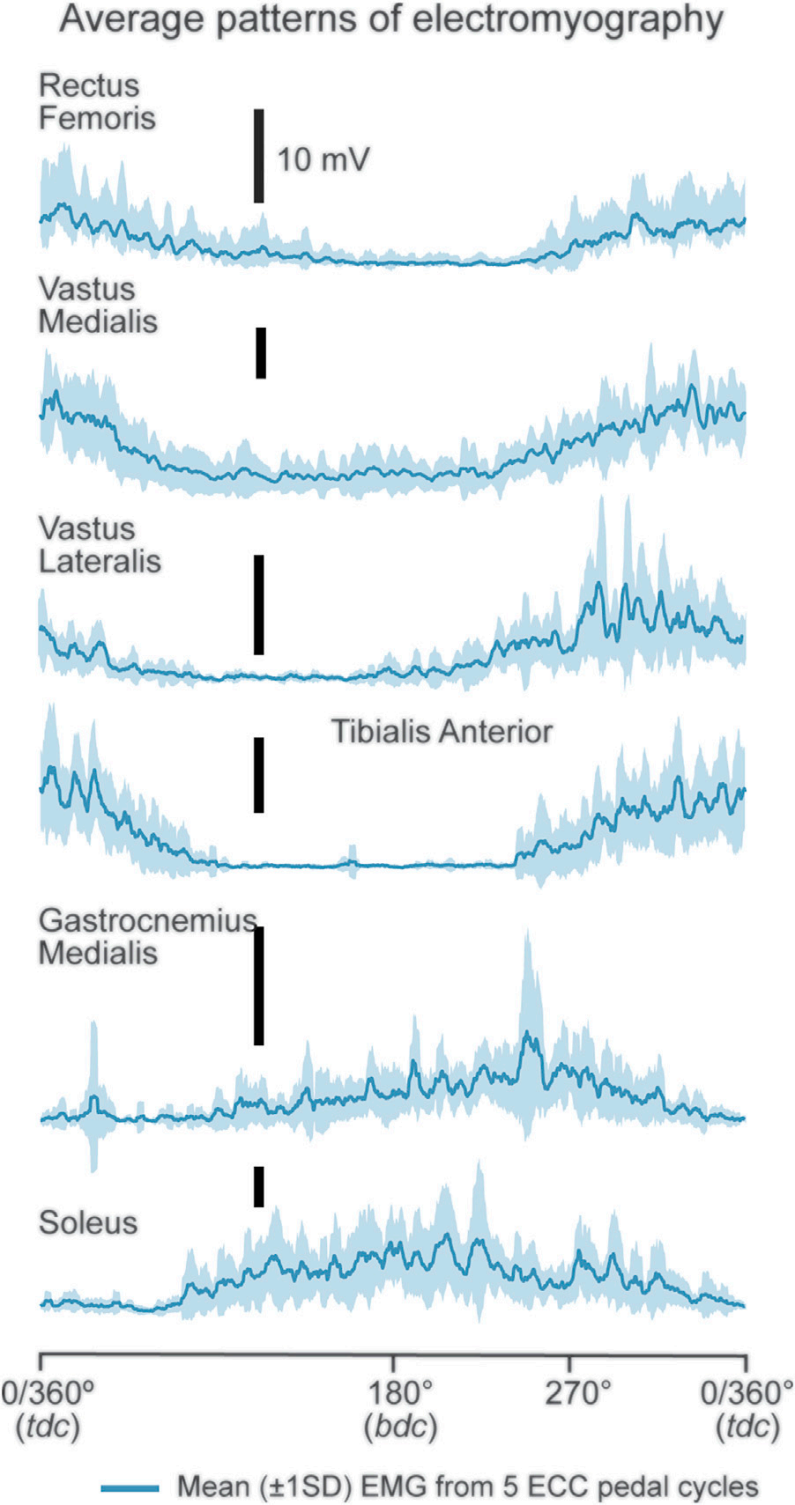
Displayed are mean (±1SD) EMG patterns from RF, VL, VM, TA, GM and SOL for a representative participant. The presented mean RMS_EMG_ trace data were binned into the following 10 s time series; (30–40 [1], 90–100 [2], 150–160 [3], 210–220 [4], 270–280 [5], 330–340 [6], 390–400 [7], 450–460 [8], 510–520 [9], 570–580 [10], 630–640 [11], 690–700 [12], 750–760 [13], 810–820 [14], 870–880 [15] s, 920–930 s [16]; [...] denotes time series number).

Processed RMS_EMG_ data were binned into 16 time series, of 10 s (s) duration, every minute after the 30th s of ECC cycling and during the final 10 s of cycling (Figure 2 caption). Ten RMS_EMG_ data points (i.e., crank position every 36°) were calculated for each revolution per time series. Crank positions within a pedal revolution were indicated by trigger pulses at 0/360° and 180° based on a pedal revolution being defined as a complete 360° backward revolution of the right pedal, rotating from top dead center (0°, TDC), beyond bottom dead center (180°) and returning to TDC (Hug et al., 2010; Walsh et al., 2021a). Data during the first 30 s of cycling was not analyzed due to the cycle ergometer ramping up to 60 rpm.

#### Reliability of RMS_EMG_ Patterns

Within-subject (intra-individual) reliability of RMS_EMG_ patterns between consecutive time series (i.e., 1v2 = 1st; 2v3 = 2nd; 3v4 = 3rd, etc.) were assessed using intra-class correlation coefficients (ICC), calculated based on mean-measures (*k* = 2), absolute agreement and two-way mixed-effects model. ICC values < 0.50, between 0.50–0.75 and 0.75–0.90 and >0.90 were considered to represent low, moderate, good and excellent reliability, respectively (Koo and Li, 2016). Standard error of measurement (SEM) and minimal detectable change (MDC) values were calculated, based on ICC values (MDC = SEM ✕ 1.96 ✕ √2) (Ries et al., 2009). Small SEM values represent better absolute reliability (Dontje et al., 2018) and MDC represent the smallest amount of change that indicates meaningful change (Ries et al., 2009).

#### Variability of RMS_EMG_ Patterns

Variance ratio (VR) was calculated (as per Equation 1), as a measure of within-subject variability (Martens et al., 2015) for all muscles at each time series (Burden et al., 2003), using RMS_EMG_ values where, *k* represents the number of RMS_EMG_ values per revolution (i.e., 10), *n* represents the number of revolutions per interval (i.e., 10), *X*_*ij*_ is the RMS_EMG_ value at the *i*th interval for the *j*th time series, and $X_{i}$ represents the mean of the RMS_EMG_ values at the *i*th interval over the *j* time series (Burden et al., 2003). A VR value of 0.40 was set as a practical upper limit (Jacobson et al., 1995), with lower VR values indicating low variability of RMS_EMG_ (Hug et al., 2008).

VR= ∑i=1k∑j=1n(Xij-Xi¯)2/k(n-1)∑i=1k∑j=1n(Xij-X¯ )2/k(n-1)with X¯= 1k∑i=1kXi¯ (1)

Coefficient of variation (CV) was also used to assess within-subject variability (Martens et al., 2015). For all muscles at each time series, CV was calculated (Equation 2) with $\chi_{i}$ representing the mean of the RMS_EMG_ values at *i*th time series and $\sigma_{i}$ is the SD of the RMS_EMG_ values about $\chi_{i}$ .

$$CV_{i}=\frac{\sigma_{i}}{\underline{\chi_{i}}}\times 100$$ (2)

#### Eccentric Muscle Coordination During Eccentric Cycling

Eccentric muscle coordination reflects the quality of muscle force modulation during ECC cycling (Vogt and Hoppeler, 2014; Kan et al., 2019) and was evaluated for each participant. Magnitude of error was calculated per time series ( ∼ 60 ECC pedal revolutions at 60 rpm) and for the duration of ECC cycling ( ∼ 900 ECC pedal revolutions at 60 rpm). Magnitude of error (%) = ((produced power output - prescribed power output) ∕ prescribed power output) (Kan et al., 2019). A small or decreasing (over time) error indicates good or improving ECC muscle coordination.

#### Determination of Familiarization

Previous studies have used reliability (i.e., ICC) and variability (i.e., CV) of RMS_EMG_ measures, as well as performance error, to determine familiarization to novel exercise tasks (Matsas et al., 2000; Calder and Gabriel, 2007; Waldron et al., 2016; Green et al., 2017). Therefore, participants were considered familiarized with ECC cycling when: 1) ICC values for the primary active primary active muscles(s) (RF, VL, VM and GM) achieved good reliability (ICC >0.75) and consistently maintained at least moderate reliability (ICC >0.50), 2) VR values for the primary active muscle(s) achieved and were consistently ≤0.40 and 3) the mean error from target power output were not significantly different.

### Statistical Analysis

All non-EMG data were tested for normality using Shapiro-Wilk tests and assumptions of sphericity. Where sphericity was violated Greenhouse-Geisser values are reported. A one-way (1 factor, *time*) repeated measures ANOVA was used to determine differences for group mean power output (relative and absolute), magnitude of error per time series, HR (absolute), %HR_max_ (as a percentage HR_max_) and CMJ heights. Main effects were compared using Bonferroni adjustments. Partial ETA squared values (η_p_^2^) were used to indicate effect size. Where data were non-normally distributed Friedman’s test with Wilcoxon signed rank used for pairwise comparisons. Kendall’s coefficient of concordance (*W*) represents effect size with interpretation based on Cohen’s interpretation guidelines (0.1 = small, 0.3 = moderate and 0.5 = large). Non-RMS_EMG_ data are presented in text as mean ± standard deviation (SD) and where specified, with ranges. All RMS_EMG_ data are presented as mean ±95% confidence intervals (CI). Data analysis was conducted using IBM SPSS Statistics for Mac, version 27 (IBM Corp., Armonk, N.Y., USA). SEM, MDC, VR and CV values were calculated using Microsoft Excel for Mac, version 16.43 (Microsoft Corp., Redmond, WA). Significance was set at *p* < 0.05.

## Results

### RPE, Perceived Exertion and Muscle Soreness

Group mean RPE, perceived exertion and muscle soreness scores showed no change during ECC cycling. Overall group mean (±SD) RPE, perceived exertion and muscle soreness scores were 7.9 ± 1.5 (range, 6–15), 4.6 ± 2.3 (range, 1–10) and 1.2 ± 1.3 (range, 0–5), respectively. These values indicate that ECC cycling was performed at a submaximal intensity.

### Power Output, HR, CMJ and Eccentric Muscle Coordination

No differences in group mean HR (*F*_3.210, 64.191_ = 1.824, *p* = 0.148, η_p_^2^ = 0.084) or %HR_max_ (*F*_3.273, 65.463_ = 1.874, *p* = 0.138, η_p_^2^ = 0.086; Figure 1B) arose during the 15 min period of ECC cycling. Similarly, mean relative (*F*_4.471, 89.429_ = 1.031, *p* = 0.400, η_p_^2^ = 0.049, Figure 1C) and absolute (*F*_3.854, 77.083_ = 1.024, *p* = 0.398, η_p_^2^ = 0.049, Figure 1C) power output values did not significantly differ during ECC cycling. Group mean (±SD) CMJ heights were also not significantly different between Pre (29.08 ± 6.85 cm), P_i_ (29.05 ± 6.75 cm) and P_30_ (29.08 ± 6.66 cm) conditions (*F*_2, 42_ = 0.007, *p* = 0.993, η_p_^2^ = 0.001) (Figure 1D).

Overall, mean magnitude of error from target power output, per time series, was 4.58 ± 1.12% (mean range of error per time series = 2.06–6.29%). Friedman’s test showed a significant difference for mean error per time series (χ^2^_14_ = 30.895, *p* = 0.006, *W* = 0.105). Wilcoxon signed-rank test showed a significant difference (*Z* = -2.677, *p* = 0.007) between the 2nd (mean = 6.01%) and 3rd (mean = 3.85%) times series (i.e., between minutes 2 and 3). No further differences in mean error from target power output were shown. These data indicate that heart rate and power output remained constant during the familiarization period, and the exercise did not affect neuromuscular status.

### Reliability of RMS_EMG_ Patterns

**FIGURE 3:**
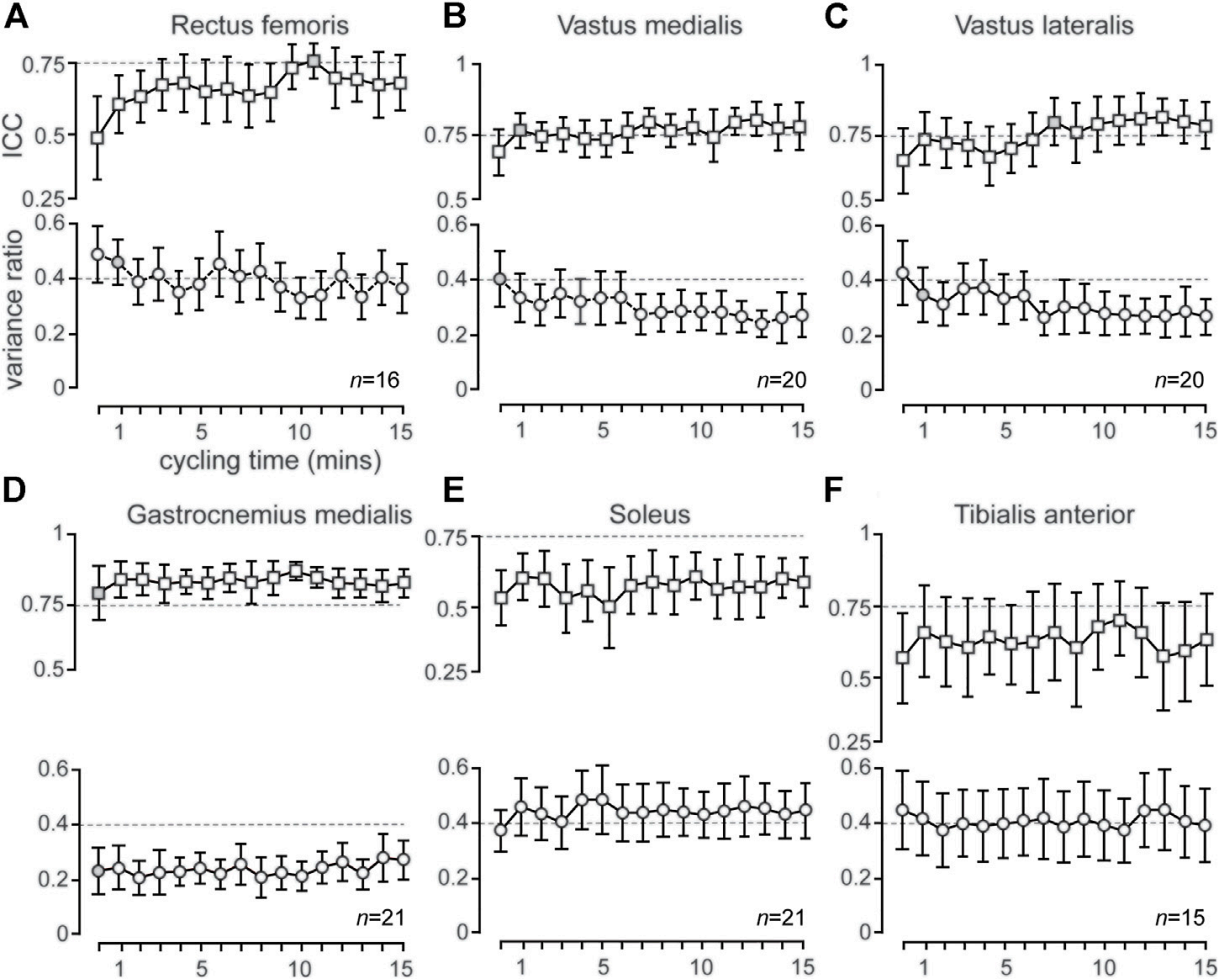
**(A–F)**: Group mean (±95% CI) ICC (squares) and VR (circles) values are presented for each consecutive time series (ICC) and time (VR). Broken lines represent ICC of 0.75 (i.e., good reliability) and VR of 0.40 (i.e., acceptable upper limit of variability), respectively. Gray shading of icons indicates the point at which the mean ICC and VR familiarization criteria were achieved for the respective muscle. *n* values (i.e., *n* = 16) represents the number of individual participants that satisfied the familiarization criteria (i.e., achieved and maintained an ICC>0.50 and VR ≤ 0.40) for the respective muscle.

Mean EMG patterns (mean of 5 pedal cycles ± 1SD) for RF, VL, VM, TA, GM and SOL are shown for a representative participant in Figure 2. Group mean (±95% CI) ICC values for RF, VM, VL, GM, SOL and TA show moderate (ICC = 0.50–0.75), good (ICC = 0.75–0.90) and excellent (ICC >0.90) reliability (Figures 3A–F; see Supplementary Table S1). The muscles primarily involved in ECC cycling (RF, VL and VM) showed an evolution in reliability over time whereas the GM, SOL and TA, despite showing moderate-excellent reliability, remained largely constant. Specifically, mean ±95% CI ICC values for the primary active muscles (RF, VL, VM) achieved good reliability (ICC = 0.75–0.90) and consistently maintained moderate reliability (ICC >0.50) from the 11v12 ( ∼ 12th minute), 8v9 ( ∼ 9th minute), and 2v3 ( ∼ 3rd minute) consecutive time series, respectively (Figures 3A–C; see Supplementary Table S1). Decreases in group mean SEM and MDC values (Supplementary Table S1), from the first to last consecutive time series (except RF), suggests absolute reliability of mean RMS_EMG_ traces improves throughout 15 min of ECC cycling.

### Variability of RMS_EMG_ Patterns

The variability of RF, VL and VM also evolved over time (as with reliability) whereas GM, SOL and TA remained constant over time. Group mean (±95% CI) VR values for RF, VL, VM, GM and TA showed acceptably low (VR ≤ 0.40) variability, with VL, VM and GM consistently maintaining mean VR values below 0.40 from the 8th, 8th and 1st time series, respectively (Figures 3A–D, Figure 3F; Table 1) Mean VR values for SOL (mean VR range = 0.37–0.49) showed higher mean variability (Figure 3E; Table 1). Coefficient of variation values for RMS_EMG_ remained consistently stable for all muscles at each time series, except for mean TA, where CV values slightly increased during ECC cycling (Table 1).

**TABLE 1:** Group mean data for VR (95% CI range), VR ranges and CV (95% CI ranges) for time series are presented for all analyzed muscles.

		Time Series															
		1	2	3	4	5	6	7	8	9	10	11	12	13	14	15	16
RF	*VR (95%CI)*	0.48 (0.38–0.58)	0.45 (0.37–0.53)	0.38 (0.30–0.46)	0.40 (0.31–0.50)	0.34 (0.26–0.41)	0.37 (0.28–0.46)	0.44 (0.33–0.56)	0.40 (0.30–0.49)	0.41 (0.32–0.51)	0.36 (0.27–0.44)	0.31 (0.24–0.39)	0.33 (0.24–0.41)	0.40 (0.32–0.48)	0.32 (0.24–0.40)	0.39 (0.29–0.49)	0.35 (0.26–0.44)
	*VR range*	0.13–0.90	0.16–0.82	0.14–0.92	0.15–0.85	0.09–0.93	0.08–0.99	0.12–0.96	0.07–0.96	0.09–0.85)	0.11–0.82	0.09–0.83	0.10–0.91	0.09–0.71	0.07–0.76	0.06–0.91	0.06–0.83
	*CV (95%CI)*	27.56 (24.12–31.01)	30.18 (25.93–34.42)	27.87 (23.97–31.76)	31.06 (24.58–37.54)	27.86 (24.53–31.18)	28.96 (24.87–33.04)	30.92 (26.64–35.21)	30.42 (26.14–34.69)	31.47 (26.84–36.09)	31.13 (25.81–36.46)	28.87 (24.36–33.37)	30.35 (24.99–35.71)	33.76 (28.60–38.91)	29.45 (24.25–34.65)	33.57 (28.82–38.33)	31.54 (25.17–37.90)
VL	*VR*	0.42 (0.30–0.54)	0.33 (0.23–0.43)	0.29 (0.21–0.37)	0.35 (0.26–0.45)	0.36 (0.26–0.46)	0.31 (0.22–0.40)	0.33 (0.24–0.42)	**0.24 (0.17–0.30)**	**0.28 (0.18–0.38)**	**0.28 (0.18–0.38)**	**0.26 (0.17–0.34)**	**0.25 (0.18–0.32)**	**0.24 (0.18–0.31)**	**0.24 (0.16–0.32)**	**0.26 (0.17–0.36)**	**0.24 (0.17–0.31)**
	*VR range*	0.06–1.00	0.08–0.93	0.13–0.85	0.06–0.86	0.08–0.99	0.05–0.91	0.08–0.83	0.06–0.68	0.09–1.09	0.09–1.01	0.06–0.91	0.07–0.76	0.11–0.80	0.07–0.80	0.07–0.81	0.06–0.60
	*CV*	33.98 (26.26–41.71)	29.60 (24.61–34.59)	27.70 (23.50–31.90)	29.04 (24.11–33.97)	30.86 (25.73–35.99)	28.38 (23.38–33.37)	30.25 (24.57–35.94)	28.52 (23.90–33.14)	30.19 (25.54–34.85)	28.89 (24.02–33.76)	28.46 (23.49–33.42)	26.92 (22.82–31.02)	27.96 (23.98–31.93)	27.07 (23.02–31.13)	28.90 (23.42–34.38)	28.65 (23.81–33.48)
VM	*VR*	0.39 (0.29–0.50)	0.32 (0.22–0.41)	0.29 (0.21–0.37)	0.33 (0.24–0.43)	0.30 (0.22–0.39)	0.32 (0.21–0.42)	0.32 (0.22–0.42)	**0.25 (0.17–0.33)**	**0.26 (0.18–0.33)**	**0.26 (0.18–0.34)**	**0.26 (0.19–0.33)**	**0.26 (0.17–0.34)**	**0.24 (0.18–0.30)**	**0.22 (0.17–0.27)**	**0.25 (0.15–0.35)**	**0.26 (0.17–0.34)**
	*VR range*	0.12–0.99	0.07–0.89	0.07–0.82	0.07–0.93	0.08–0.88	0.10–0.98	0.09–1.01	0.06–0.91	0.10–0.78	0.09–0.79	0.09–0.73	0.07–0.89	0.09–0.64	0.08–0.44	0.09–0.95	0.08–0.77
	*CV*	32.08 (27.05–37.12)	29.61 (24.36–34.86)	27.48 (23.39–31.58)	31.25 (26.07–36.43)	30.93 (24.63–37.22)	30.23 (25.76–34.70)	30.55 (24.97–36.12)	28.37 (24.13–32.61)	29.35 (24.66–34.04)	28.56 (23.67–33.45)	28.23 (23.71–32.76)	28.16 (23.05–33.26)	26.48 (22.09–30.88)	24.83 (21.32–28.34)	26.34 (22.86–29.82)	27.10 (22.03–32.17)
SOL	*VR*	0.37 (0.30–0.45)	0.46 (0.36–0.56)	0.44 (0.34–0.53)	0.40 (0.31–0.50)	0.49 (0.39–0.59)	0.49 (0.37–0.61)	0.44 (0.34–0.54)	0.44 (0.34–0.54)	0.45 (0.36–0.55)	0.44 (0.36–0.53)	0.43 (0.35–0.51)	0.45 (0.35–0.54)	0.47 (0.36–0.57)	0.46 (0.37–0.55)	0.43 (0.35–0.52)	0.45 (0.35–0.55)
	*VR range*	0.07–0.69	0.08–1.00	0.09–1.01	0.13–0.97	0.09–0.96	0.08–1.03	0.07–0.94	0.07–0.86	0.09–0.92	0.13–0.82	0.09–0.85	0.11–0.93	0.09–0.96	0.08–0.85	0.16–0.75	0.10–0.97
	*CV*	22.24 (18.38–26.11)	22.14 (17.96–26.32)	21.06 (17.77–24.34)	20.68 (17.57–23.78)	23.19 (18.81–27.57)	22.62 (18.63–26.61)	22.38 (18.05–26.71)	22.72 (18.47–26.97)	23.09 (18.76–27.42)	22.78 (18.11–27.44)	20.03 (16.27–23.80)	21.56 (17.87–25.25)	21.58 (18.22–24.94)	20.94 (16.98–24.91)	21.32 (18.14–24.50)	20.94 (17.52–24.37)
GM	*VR*	**0.23 (0.15–0.31)**	**0.24 (0.17–0.32)**	**0.20 (0.14–0.26)**	**0.22 (0.15–0.30)**	**0.23 (0.18–0.28)**	**0.24 (0.18–0.29)**	**0.22 (0.17–0.27)**	**0.25 (0.18–0.33)**	**0.20 (0.13–0.28)**	**0.22 (0.16–0.28)**	**0.21 (0.16–0.26)**	**0.24 (0.19–0.30)**	**0.26 (0.20–0.33)**	**0.22 (0.16–0.27)**	**0.28 (0.19–0.36)**	**0.27 (0.20–0.34)**
	*VR range*	0.05–0.97)	0.08–0.94	0.06–0.59	0.07–0.92	0.09–0.55	0.05–0.59	0.07–0.53	0.06–0.74	0.06–0.83	0.09–0.58	0.07–0.55	0.06–0.59	0.05–0.77	0.06–0.55	0.08–0.71	0.06–0.63
	*CV*	27.38 (23.32–31.43)	29.83 (25.87–33.79)	29.29 (23.93–34.65)	31.54 (26.89–36.20)	32.33 (28.42–36.25)	33.61 (28.20–39.01)	33.02 (27.69–38.35)	35.74 (29.97–41.51)	30.24 (25.50–34.98)	35.32 (28.52–42.12)	29.53 (25.83–33.23)	33.11 (28.67–37.56)	33.65 (28.65–38.65)	31.91 (26.12–37.70)	34.79 (28.88–40.70)	33.86 (27.69–40.03)
TA	*VR*	0.45 (0.31–0.58)	0.41 (0.28–0.54)	0.37 (0.24–0.50)	0.40 (0.28–0.51)	0.39 (0.26–0.51)	0.39 (0.27–0.52)	0.40 (0.28–0.52)	0.41 (0.27–0.55)	0.38 (0.26–0.51)	0.41 (0.28–0.54)	0.39 (0.26–0.51)	0.37 (0.26–0.48)	0.45 (0.32–0.58)	0.45 (0.30–0.59)	0.40 (0.28–0.53)	0.39 (0.26–0.52)
	*VR range*	0.07–1.03	0.08–1.01	0.06–0.97	0.06–0.91	0.09–0.99	0.07–0.98	0.05–0.89	0.10–1.01	0.08–1.02	0.07–0.96	0.06–0.95	0.11–0.85	0.06–1.02	0.11–0.98	0.11–1.00	0.08–0.95
	*CV*	24.15 (19.21–29.09)	27.17 (21.24–33.11)	26.44 (20.87–32.01)	29.06 (22.15–35.96)	31.13 (23.68–38.58)	27.63 (22.43–32.83)	26.90 (22.14–31.66)	30.50 (23.48–37.52)	32.31 (25.78–38.84)	28.12 (22.89–33.35)	29.07 (23.89–34.25)	28.82 (23.36–34.27)	35.29 (26.71–43.87)	30.48 (25.37–35.58)	34.10 (26.25–41.95)	29.88 (24.54–35.22)

Bolded text represents the time series when mean VR values satisfied the familiarization criteria (achieved and maintained a VR ≤ 0.40) for the respective muscles. Abbreviations: CI, confidence interval; CV, coefficient of variation; GM, medial gastrocnemius; RF, rectus femoris; SOL, soleus; TA, tibialis anterior; VL, vastus lateralis; VM, vastus medialis; VR, variance ratio.

**FIGURE 4:**
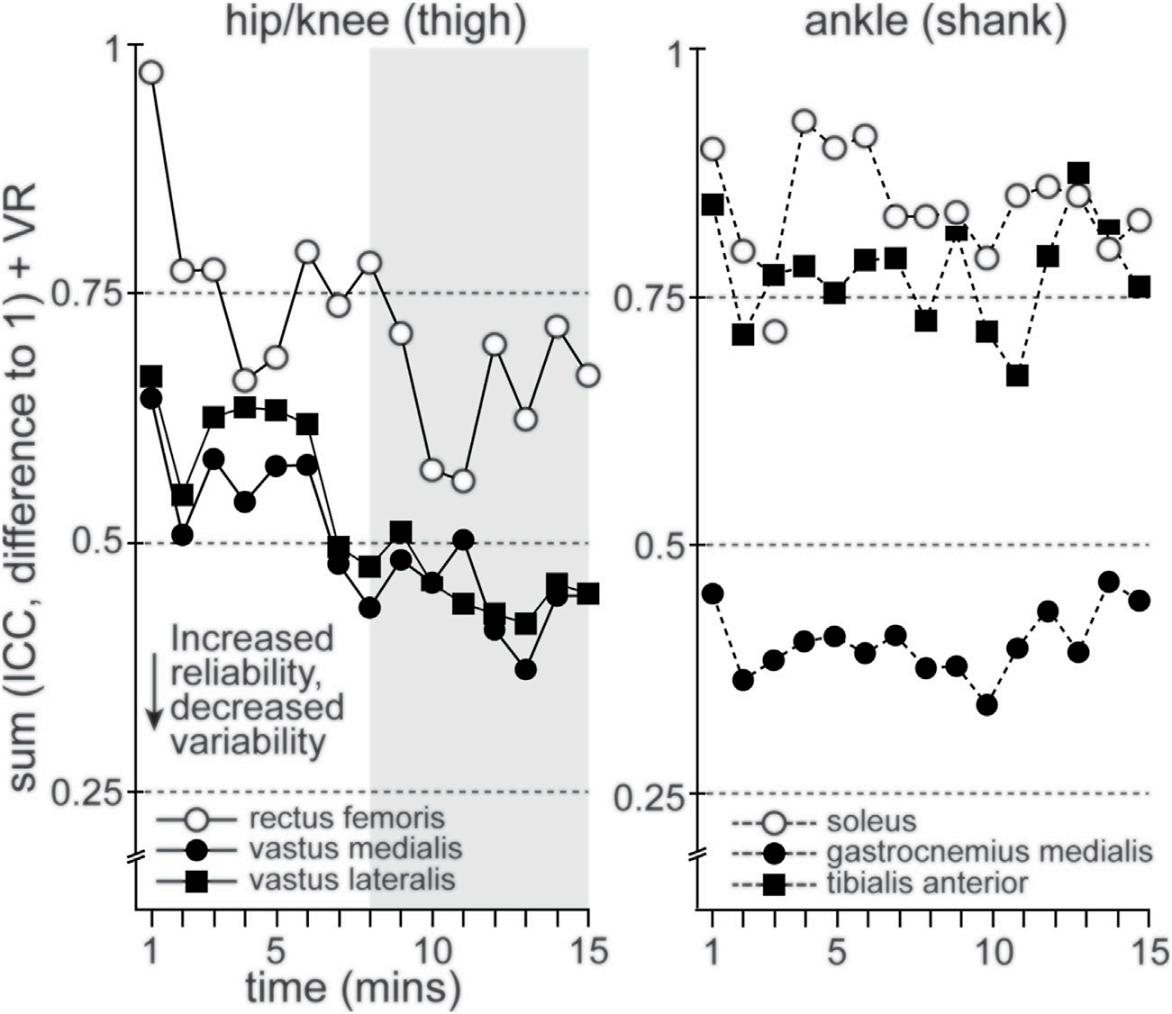
Plots the summation of group mean ICC (difference from 1.0) and VR values, based on joint articulation (hip/knee and ankle) during 15 min of ECC cycling, to contextualize the mean time to familiarization. RF, VL and VM muscles (hip/knee joint) show a descending trend, representing improved reliability with decreased variability.

In order to pair measures of reliability and variability, we added the difference between ICC values and 1.0 (measure of the difference from ICC values of 1.0) and VR measures. A trend towards a lowering of values would indicate increased reliability and decreased variability. These values are shown in Figure 4 for RF, VM, VL, SOL, GM and TA. There was a clear trend in RF, VM and VL towards much lower values after the 12th time series or 690–700 s. No clear trend could be seen in SOL, GM or TA over the period, despite GM showing acceptable reliability and variability (see above). Overall, all participants were considered familiarized with ECC cycling by the 15th time series (870–880 s) or 14:30–14:40 min of ECC cycling (Figure 4).

## Discussion

To our knowledge, this is the first study to determine single-session familiarization to ECC cycling, using reliability and variability of lower limb muscle activation patterns, among naïve participants. In support of the hypotheses, all participants produced reliable muscle activation patterns, of acceptably low variability, while accurately maintaining their prescribed target power output. These findings indicate that all 22 naïve participants were able to familiarize with ECC cycling, based on satisfying the aforementioned familiarization criteria, within a 15 min duration. Specifically, by the 15th time series (i.e., 870–880 s) RMS_EMG_ patterns for the primary active muscles 1) achieved good reliability (ICC >0.75) and consistently maintained at least moderate reliability (ICC >0.50), 2) achieved and consistently maintained a VR ≤ 0.40 for most of the primary active muscles and lastly, that 3) mean error from target power output was not significantly different after the 3rd minute of ECC cycling. Moreover, there was a clear difference in the evolution of reliability and variability for VL and VM, compared to GM (Figure 4).

The current findings complement previous studies suggesting that familiarization to maximal recumbent ECC cycling requires a single practice session (Green et al., 2017). These authors (Green et al., 2017), suggest that pedaling technique improves as participants familiarized with maximal ECC cycling. This reflects improved reliability of RF and VL muscle activation patterns achieved following a single familiarization session (Green et al., 2017) and coincides with the significant absorption of power by the knee extensors during ECC cycling (Elmer et al., 2010). Similarly, reductions in RMS_EMG_ activity of RF, VL and SOL reportedly occurred following four variable-intensity, short-duration (2 × 1–1.5 min) submaximal ECC cycling sessions (Clos and Lepers, 2020). Reduced lower leg muscle activity in RF, VL and SOL has been linked to adaptations occurring from the repeated bout effect (Clos and Lepers, 2020) that selectively reduces specific motor unit activity (Enoka, 1996; McHugh, 2003), possibly through increased spinal inhibition during ECC contractions (Behrens, 2017). This explanation may well account for the improved muscle activation patterns observed in the current study. Additionally, muscle control strategies of the lower limb appear to adapt to a novel cycling task (i.e., asymmetrical cycling) within 10 min, due to feedforward and feedback modifications (Zych et al., 2018), further supporting the longer familiarization duration used in this study. Indeed, the current muscle activations patterns likely refine due to continual biofeedback afforded when completing a rhythmic cycling task, over an extended timeframe (Torricelli et al., 2020). Therefore, the current findings support the assumption that familiarization occurs during 15 min of ECC cycling at the prescribed experimental workload (Clos et al., 2022).

Previous studies have reported decreased RMS_EMG_ activity, both within and across several ECC cycling sessions for VL (Bigland-Ritchie and Woods, 1976; Dufour et al., 2007; LaStayo et al., 2008; Peñailillo et al., 2013; Lechauve et al., 2014; Peñailillo et al., 2017; Clos and Lepers, 2020), VM (Dufour et al., 2007) and RF (Dufour et al., 2007; Peñailillo et al., 2017; Clos and Lepers, 2020) despite differing from the current study with respect to cycling intensity (Dufour et al., 2007; LaStayo et al., 2008; Lechauve et al., 2014), time (< or >15 min) (LaStayo et al., 2008; Peñailillo et al., 2013; Lechauve et al., 2014; Peñailillo et al., 2017; Clos and Lepers, 2020), number of sessions (>1) (Bigland-Ritchie and Woods, 1976; LaStayo et al., 2008; Peñailillo et al., 2013; Lechauve et al., 2014; Peñailillo et al., 2017; Clos and Lepers, 2020) or when comparing modalities (concentric *vs*. ECC) (Bigland-Ritchie and Woods, 1976; Dufour et al., 2007; Peñailillo et al., 2017; Clos and Lepers, 2020). These findings corroborate our measures of low variability (i.e., VR) in RMS_EMG_ for RF, VL, VM, GM and SOL in the current study. This low variability also corresponded to high mean reliability (i.e., ICC, SEM, MDC) for RF, VL, VM, GM and SOL across consecutive time series. Interestingly, our low VR values for VL, VM, and GM are comparable to those reported among trained cyclists performing submaximal concentric cycling (Hug et al., 2008). Furthermore, variability of RF and TA is substantially lower in the current study. Taken together, 15 min of novel ECC cycling enables naïve participants to produce lowly variable muscle activation patterns, comparable to that of trained cyclists performing submaximal concentric cycling.

It is worth noting, however, the respective difference in the evolution of how these muscles achieve acceptable reliability and variability. Figure 4 demonstrates a clear pattern of improvement (i.e., increasing reliability and decreasing variability) in RF, VL and VM during 15 min of ECC cycling. In comparison, GM, SOL and TA show no such improvement. This difference may relate to the actions of the specific muscle groups and their respective joint articulation. Indeed, RF, VL and VM (i.e., knee extensors) work to primarily absorb and transfer power during cycling, including ECC cycling (Hug et al., 2008; Elmer et al., 2010; Hug et al., 2010). Comparatively, muscles articulating about the ankle (i.e., plantar and dorsiflexors) absorb less power (10% at ankle versus 58% at knee) during ECC cycling (Elmer et al., 2010; Green et al., 2017; Penailillo et al., 2017). Therefore, GM, SOL and TA more likely act, through co-contraction, to stabilize the pedal to allow for absorption and transfer of power during ECC cycling. Furthermore, it should be noted that this study was conducted using an ECC cycle ergometer instrumented to ensure muscle contraction was isolated to the opposing phase of ECC cycling (Walsh et al., 2021a). Subsequently, familiarization, based on stabilization of muscle activations patterns, may require more time when ECC contractions are not specifically controlled during ECC cycling.

Consistent reliability and low variability of RMS_EMG_ for RF, VL, VM, GM and SOL is analogous with consistently low error from target power output after the 3rd time series (150–160 s) of ECC cycling. Furthermore, RPE, perceived exertion, muscle soreness and %HR_max_ values (participant range 29–76%) suggest that ECC cycling, at 10% PETP, was of low-moderate intensity (i.e., submaximal) and comparable with power outputs prescribed in previous ECC cycling studies (Walsh et al., 2021b). Moreover, neuromuscular status and objective lower limb fatigue (Sanchez-Medina and González-Badillo, 2011; Balsalobre-Fernández et al., 2014; Claudino et al., 2017) were unaffected, based on no difference in CMJ heights Pre or Post ECC cycling. Compared to these findings, previously reported magnitudes of error from target torque, across two bouts of semi-recumbent ECC cycling, were substantially greater (mean range of error = 19.4–26.1%) (Kan et al., 2019). These authors (Kan et al., 2019) suggested that an inability to maintain a target output relates to the complexity of ECC cycling that requires more sustained concentration to perform, compared to concentric cycling (Mueller et al., 2009; Hoppeler, 2014). However, current participants were able to consistently match prescribed target outputs by the 3rd time series (150–160 s), despite no previous familiarization. This could be due to differences between ECC cycle ergometers used in these studies. Of note is the difference in target outputs between the current study, being power output (W, W/kg^−1^) and that of Kan et al. (2019) (Kan et al., 2019), being torque (Nm). However, given that power output is derived from torque, comparison between the studies is considered valid.

There are two main limitations of this study. Firstly, the current study would have benefitted from recording muscle activation patterns from gluteus maximus, a primary hip extensor, given that the hip joint, along with the knee and ankle joints, absorbs power during semi-recumbent ECC cycling (Elmer et al., 2010). RMS_EMG_ data recorded from gluteus maximus would have provided additional insight into the neuromuscular adaptations occurring at the hip during familiarization to ECC cycling. Secondly, the current study did not examine between-session repeatability. Investigating between-session repeatability would have provided further insight into participant familiarization and should be considered in further studies.

Based on these findings, researchers and clinicians applying submaximal ECC cycling protocols can familiarize naïve participants within a single 15 min session. Providing naïve participants sufficient time (i.e., 15 min) to familiarize with novel ECC cycling will likely minimize variability and subsequently, improve the reliability of recorded measures (Matsas et al., 2000; Bischoff et al., 2012) particularly during ECC cycling (Green et al., 2017; Penailillo et al., 2017). Furthermore, a single-session familiarization protocol reduces time constraints associated with multi-visit familiarization protocols.

## Conclusion

In conclusion, the current study confirms that naïve participants familiarize with ECC cycling, during a single 15-min session. The currently proposed familiarization protocol is arguably more robust than previous protocols that assume familiarization (Peñailillo et al., 2015; Penailillo et al., 2017; Rakobowchuk et al., 2018; Kan et al., 2019; Pageaux et al., 2020; Clos et al., 2022) and could be easily implemented by future studies in lieu of previous, less-specific procedures used to infer familiarity among naïve participants. Therefore, it is recommended that future studies, implementing similar submaximal ECC cycling protocols, familiarize naïve participants for 15 min at the prescribed experimental workload. On-going studies that adequately familiarize participants with submaximal ECC cycling, are likely to produce more reliable measurements and therefore, better realize the application of subsequent findings (Green et al., 2017). Lastly, the current findings are relative to healthy participants performing submaximal ECC cycling. Whether other cohorts, including clinical and elderly populations, can familiarize with submaximal ECC cycling during a single session, is unknown and requires future investigation.

## Data Availability

The raw data supporting the conclusions of this article will be made available by the authors, without undue reservation.
