# Supplementary material for: Reliability and Variability of Lower Limb Muscle Activation as Indicators of Familiarity to Submaximal Eccentric Cycling
Source: Front Physiol. 2022 Jul 8;13:953517. doi: 10.3389/fphys.2022.953517 (PMC9304807; doi:10.3389/fphys.2022.953517)
Supplement: Supplementary file 1 [file Table1.DOCX]

**Supplementary Material**

Table. Group mean data for ICC (95% CI range), SEM and MDC for consecutive time series are presented for all analyzed muscles. Bolded text represents the consecutive time series when mean ICC values satisfied the familiarization criteria (achieved good reliability [ICC = 0.75-0.90] and maintained at least moderate reliability [0.50-0.75]) for the respective muscles.

|  |  | **Consecutive Time Series** | | | | | | | | | | | | | | |
| --- | --- | --- | --- | --- | --- | --- | --- | --- | --- | --- | --- | --- | --- | --- | --- | --- |
|  |  | 1v2 | 2v3 | 3v4 | 4v5 | 5v6 | 6v7 | 7v8 | 8v9 | 9v10 | 10v11 | 11v12 | 12v13 | 13v14 | 14v15 | 15v16 |
| **RF** | *ICC (95%CI)* | 0.48 (0.32-0.63) | 0.60 (0.50-0.71) | 0.63 (0.54-0.73) | 0.67 (0.58-0.77) | 0.68 (0.57-0.79) | 0.65 (0.53-0.77) | 0.66 (0.54-0.78) | 0.63 (0.52-0.75) | 0.65 (0.54-0.75) | 0.74 (0.66-0.82) | **0.76 (0.70-0.83)** | **0.70 (0.59-0.81)** | **0.70 (0.61-0.78)** | **0.67 (0.55-0.79)** | **0.68 (0.58-0.78)** |
|  | *SEM* | 5.83 | 4.68 | 4.82 | 5.73 | 4.33 | 4.79 | 4.99 | 5.67 | 5.13 | 4.86 | 4.54 | 4.97 | 5.19 | 4.27 | 5.11 |
|  | *MDC* | 11.74 | 10.02 | 10.07 | 10.39 | 10.01 | 11.47 | 11.51 | 11.85 | 11.62 | 11.97 | 12.35 | 12.15 | 11.79 | 10.83 | 10.57 |
| **VL** | *ICC* | 0.66 (0.54-0.78) | 0.74 (0.65-0.84) | 0.73 (0.64-0.82) | 0.72 (0.64-0.80) | 0.68 (0.57-0.79) | 0.71 (0.62-0.80) | 0.74 (0.64-0.84) | **0.81 (0.72-0.89)** | **0.78 (0.66-0.87)** | **0.80 (0.70-0.90)** | **0.81 (0.72-0.90)** | **0.82 (0.72-0.91)** | **0.82 (0.76-0.89)** | **0.80 (0.73-0.88)** | **0.79 (0.71-0.88)** |
|  | *SEM* | 3.53 | 2.95 | 2.99 | 3.19 | 3.28 | 3.24 | 3.53 | 3.60 | 3.84 | 2.93 | 3.12 | 2.82 | 2.69 | 3.26 | 3.26 |
|  | *MDC* | 9.79 | 8.19 | 8.27 | 8.85 | 9.09 | 8.97 | 9.78 | 9.98 | 10.65 | 8.12 | 8.66 | 7.80 | 7.46 | 9.05 | 9.03 |
| **VM** | *ICC* | 0.67 (0.56-0.79) | **0.78 (0.69-0.87)** | **0.75 (0.68-0.82)** | **0.76 (0.67-0.85)** | **0.74 (0.65-0.83)** | **0.74 (0.65-0.83)** | **0.77 (0.67-0.87)** | **0.82 (0.75-0.89)** | **0.78 (0.70-0.86)** | **0.80 (0.71-0.89)** | **0.75 (0.62-0.89)** | **0.83 (0.76-0.89)** | **0.85 (0.81-0.89)** | **0.80 (0.71-0.89)** | **0.81 (0.71-0.91)** |
|  | *SEM* | 6.24 | 4.85 | 4.65 | 5.14 | 5.01 | 4.65 | 5.25 | 5.43 | 5.15 | 4.21 | 4.64 | 4.31 | 3.84 | 3.73 | 3.22 |
|  | *MDC* | 14.48 | 10.41 | 10.54 | 10.66 | 11.64 | 12.15 | 11.78 | 11.20 | 13.24 | 10.10 | 11.80 | 10.35 | 9.88 | 7.80 | 8.00 |
| **SOL** | *ICC* | 0.56 (0.47-0.66) | 0.64 (0.56-0.72) | 0.63 (0.54-0.73) | 0.56 (0.44-0.68) | 0.59 (0.49-0.69) | 0.53 (0.39-0.67) | 0.61 (0.51-0.71) | 0.62 (0.51-0.73) | 0.61 (0.51-0.71) | 0.64 (0.56-0.72) | 0.60 (0.50-0.70) | 0.60 (0.49-0.72) | 0.60 (0.50-0.71) | 0.64 (0.57-0.70) | 0.62 (0.54-0.71) |
|  | *SEM* | 1.91 | 1.61 | 1.39 | 1.65 | 1.50 | 1.59 | 1.91 | 1.64 | 1.70 | 1.70 | 1.58 | 1.29 | 1.39 | 1.39 | 1.29 |
|  | *MDC* | 6.19 | 5.21 | 5.01 | 5.68 | 5.58 | 5.97 | 6.33 | 5.59 | 5.91 | 5.75 | 5.38 | 5.16 | 5.30 | 4.92 | 5.33 |
| **GM** | *ICC* | **0.79 (0.70-0.88)** | **0.84 (0.78-0.90)** | **0.84 (0.78-0.89)** | **0.82 (0.76-0.89)** | **0.83 (0.79-0.87)** | **0.83 (0.77-0.88)** | **0.85 (0.80-0.89)** | **0.83 (0.76-0.90)** | **0.84 (0.79-0.90)** | **0.87 (0.84-0.90)** | **0.85 (0.81-0.88)** | **0.83 (0.78-0.88)** | **0.82 (0.78-0.87)** | **0.82 (0.76-0.87)** | **0.83 (0.78-0.87)** |
|  | *SEM* | 3.95 | 3.20 | 3.24 | 3.75 | 3.72 | 3.31 | 3.53 | 3.40 | 3.27 | 3.15 | 3.15 | 3.40 | 3.11 | 3.24 | 3.22 |
|  | *MDC* | 11.26 | 10.06 | 8.80 | 10.51 | 11.58 | 10.33 | 10.32 | 9.81 | 9.73 | 10.66 | 9.22 | 10.59 | 9.95 | 9.82 | 10.30 |
| **TA** | *ICC* | 0.57 (0.43-0.71) | 0.66 (0.52-0.80) | 0.62 (0.49-0.76) | 0.61 (0.45-0.76) | 0.64 (0.52-0.76) | 0.61 (0.49-0.74) | 0.62 (0.47-0.78) | 0.66 (0.51-0.80) | 0.60 (0.42-0.77) | 0.67 (0.54-0.80) | 0.70 (0.59-0.81) | 0.65 (0.52-0.79) | 0.57 (0.41-0.74) | 0.59 (0.44-0.74) | 0.63 (0.49-0.77) |
|  | *SEM* | 2.82 | 2.64 | 3.92 | 2.53 | 2.74 | 2.13 | 2.17 | 2.36 | 2.34 | 1.98 | 2.13 | 2.50 | 2.37 | 2.88 | 2.12 |
|  | *MDC* | 7.59 | 6.34 | 7.07 | 8.00 | 7.91 | 6.28 | 6.51 | 6.83 | 7.02 | 6.02 | 6.74 | 6.75 | 7.59 | 8.10 | 6.24 |

Abbreviations: CI = Confidence interval, GM = Medial gastrocnemius, ICC = Intra-class correlation coefficient, MDC = Minimal detectable change, RF = Rectus Femoris, SEM = Standard error of measurement, SOL = Soleus, TA = Tibialis Anterior, VL = Vastus Lateralis, VM = Vastus Medialis.
